# UK dietitians' attitudes and experiences of formula very low‐ and low‐energy diets in clinical practice

**DOI:** 10.1111/cob.12509

**Published:** 2022-01-23

**Authors:** Adrian Brown, Naomi Brosnahan, Dorsa Khazaei, Jed Wingrove, Stuart W. Flint, Rachel L. Batterham

**Affiliations:** ^1^ Centre for Obesity Research University College London London UK; ^2^ Bariatric Centre for Weight Management and Metabolic Surgery University College London Hospital NHS Trust London UK; ^3^ National Institute of Health Research UCLH Biomedical Research Centre London UK; ^4^ School of Medicine, Dentistry & Nursing University of Glasgow Scotland UK; ^5^ Counterweight Ltd London UK; ^6^ School of Psychology University of Leeds Leeds UK; ^7^ Scaled Insights, Nexus University of Leeds Leeds UK

**Keywords:** attitudes, dietitians, formula diets, obesity, type 2 diabetes remission, very low/low‐energy diets

## Abstract

Despite evidence that formula very low‐energy diets (VLED) and low‐energy diets (LED) are both effective and safe as treatments for obesity and type 2 diabetes, these diets remain underutilized in the United Kingdom. The aim of this study was to explore UK dietitians' attitudes and experiences of using formula VLED and LED. A cross‐sectional survey was disseminated between September 2019 and April 2020 through websites, social media platforms and dietetic networks using snowball sampling. In total, 241 dietitians responded to the online survey with 152 participants included in the final analysis (female [94.1%], mean age 40.8 years [SD 9.5]; median 12 years [interquartile range 8, 22] within dietetic practice). One hundred and nine (71.7%) participants reported currently using VLED/LED in clinical practice and 43 (28.3%) did not. Those with lower motivation and confidence in implementing VLED/LED in clinical practice were less likely to use them. Cost and adherence were the two highest reported barriers to use. Dietitians perceived VLED/LED were effective, but concerns remained about long‐term effectiveness, particularly for some patient groups. Dietitians also reported that further education, funding and service infrastructure, including access to clinic space and administrative support, were required to help embed VLED/LED into routine clinical practice. With clinical services now regularly offering VLED/LED programmes in the United Kingdom, dietitians are ideally placed to provide long‐term support. However, understanding, reporting and addressing the potential barriers (funding/infrastructure and education) appear to be key requirements in increasing the delivery of VLED/LED programmes nationally.

**What is already known about this subject**

- Formula very low‐energy diets (VLED) and low‐energy diets (LED) are efficacious and safe for the management of both obesity and type 2 diabetes within clinical practice in the United Kingdom.
- Despite this VLED/LED remain underutilized in the United Kingdom and there is a lack of understanding about the experiences and attitudes of dietitians who use them.

**What this study adds**

- Dietitians reported using VLED/LED frequently in clinical practice and perceived they were effective, but concerns remained about long‐term effectiveness. The most commonly reported barriers of VLED/LED use were cost and adherence.
- Dietitians reported that further education, funding and infrastructure (i.e., access to clinic space, administrative support) were essential to help embed VLED/LED into clinical practice.
- The information from this study can be used by services managers, commissioners and dietitians to help embed VLED/LED programmes as a treatment option for dietitians to use with patients.

## 1 INTRODUCTION

In 2016, it was estimated there were approximately 650 million people living with obesity globally.^1^ Obesity is associated with an increased risk of type 2 diabetes (T2D), heart disease, stroke and some cancers^1^ and has resulted in worldwide focus. Despite this, there remains a paucity of interventions that have been shown to be effective in reducing the prevalence of obesity. Therefore, effective treatments for obesity and its related co‐morbidities continue to be key priorities globally.

Formula very low‐energy diets and low‐energy diets (VLED/LED) are specially formulated products, that come in the form of liquid soups, shakes or porridges, as well as bars.^2^ VLED are defined as having ≤800 kcal (3300 kJ) and LED between 800 and 1200 kcal (3351–5021 kJ).^2^ There is now increasing evidence that using formula VLED/LED results in clinically significant weight loss and T2D remission.^2^, ^3^, ^4^, ^5^ Weight loss of between 10% and 15% has been reported within the initial total diet replacement (TDR) phase,^6^, ^7^ and have been shown to be maintained for up to 4 years.^2^, ^8^ Despite substantial scientific evidence and recent UK Government support for the use of LED,^9^, ^10^ both formula VLED/LED are infrequently used in dietetic practice and issues concerning their use persist including poor adherence, potential side effects and long‐term efficacy.^11^

From the limited data on the use of VLED in practice, all of which have originated from Australia, VLED/LED are only offered to patients by 3%–7% of healthcare professionals (HCP), including dietitians, as a weight‐loss treatment.^12^, ^13^ As such, the views and perceptions of VLED/LED held amongst dietitians in the United Kingdom remain unknown despite them often being the primary HCP delivering these interventions. Given that a national pilot is currently being delivered by NHS England for their use in T2D remission,^9^ it is essential that UK dietitians feel equipped to deliver such programmes.

The aim of this study was to explore dietitians' attitudes and experiences of using formula VLED and LED in current clinical practice in the United Kingdom.

## 2 METHODS

Dietitians were recruited through online advertisement to complete an online cross‐sectional survey between August 2019 and March 2020. Recruitment invitations were disseminated through the British Dietetic Association (BDA) website, social media advertisement including Facebook, Twitter and dietetic networks using snowball sampling. Potential participants gained access to the survey through a link using University College London (UCL) Opinio platform. Eligible participants were required to be a registered dietitian and aged between 20 and 65 years.

All participants were provided with an electronic participant information sheet and gave informed consent (electronically) prior to participating. After which participants provided demographic data including age, gender, country of residency, years since dietitian registration and the area of clinical practice they worked in. The survey comprised of the following three sections and included both open and closed questions:

1. Attitudes and experiences of dietitians using formula VLED/LED in current practice.
2. Attitudes and experiences of delivery protocols at the different stages of the formula VLED/LED programmes, e.g. TDR, reintroduction of food and weight loss maintenance, including the use of rescue packages (reusing a VLED/LED for up to 4 weeks to treat weight regain) to correct weight regain.
3. Motivation, confidence and barriers experienced by dietitians when implementing formula VLED/LED in current practice.

Participants were asked to scale their understanding, confidence and motivation using a 10‐point Likert scale from ‘1’ = least to ‘10’ = most. The study was granted ethical approval by the UCL Research Ethics Committee (REC number 16191/003). A copy of the survey questions is provided in Appendix S1.

### 2.1 Data analysis

Descriptive data were summarized using means (standard deviation [SD]) for continuous variables depending on distribution. Categorical data were reported using counts (percentages). Normality tests were used to assess the distribution of the continuous data, and where data were not normally distributed data were presented as median (interquartile range [IQR]). All statistical analyses were conducted using SPSS version 24 with statistical significance being defined as a *p*‐value <.05.

Differences between baseline characteristics were analysed using either independent sample *t*‐test or Mann–Whitney *U* test for continuous data and chi‐squared for categorical data. Correlations between categorical data and motivation and confidence were assessed using Spearman's correlation coefficient.

Binomial and ordinal logistic remission alongside generalized linear models were used to identify factors that predicted understanding of formula VLED/LED, views on long‐term weight loss and confidence and motivation of formula VLED/LED implementation. Covariates within the models were age, years of registration, current use of formula diet or not in practice and frequency of use in practice. Odds ratios, 95% confidence intervals (CI) and *p*‐values were reported for ordinal and binomial logistic regression.

To explore predictors of beliefs of long‐term weight loss, the question asking ‘Do you believe that VLED/LED can achieve long term weight loss’ was made into a dichotomous variable, where the responses, ‘No’ and ‘Maybe’, were added together to create the variable ‘No’ (0) while ‘Yes’ remained the same (1).

Qualitative data were collected using four open‐ended free‐text questions to gather greater insights into dietitians' views and experience of using formula VLED/LED (see Appendix S1 for the five open‐ended questions). Three authors independently coded the five open‐ended questions (AB, NB, SWF) to identify the key themes and subthemes. Thereafter, through discussion, which included resolving any coding disagreements, the final themes, subthemes and supporting quotes were agreed.

## 3 RESULTS

### 3.1 Study population and baseline demographics

A total of 241 dietitians responded to the survey. Participants who did not give consent (*n* = 50), did not complete 60% of the survey (*n* = 21) or did not practice dietetics in the United Kingdom (*n* = 18) were excluded leaving a total of 152 (63%) participants in the final analysis.

Most participants were female (*n* = 143, [94.1%]), with a mean age of 40.8 (SD 9.5) years and had been registered dietitians for a median of 12 years (IQR 8, 22). Over half of the participants resided in England (*n* = 99, [59.2%]) and 38 (25%) from Scotland. Regarding the areas of practice that participants worked in, 90 (59.2%) reported working in weight management and over half (*n* = 77 [50.7%]) worked in diabetes care (Table 1), with 64 (42.1%) working in two or more areas of clinical practice. Table 1 summarizes the demographic characteristics.

**TABLE 1:** Demographic characteristics of the participants (*n* = 152)

Characteristic		
Gender	Female	143 (94.1)
	Male	9 (5.9)
Age, median (IQR)	Years	39.5 (34–49)^a^
Country of residency in the United Kingdom	England	99 (65.1)
	Scotland	38 (25.0)
	Wales	14 (9.2)
	Northern Ireland	1 (0.7)
Years since registration median (IQR)	Years	12 (8–22)^a^
Area of clinical practice^b^	Weight management	90 (59.2)
	Diabetes	77 (50.7)
	Bariatric Surgery	40 (26.3)
	Endocrinology	5 (3.3)
	Paediatrics	2 (1.3)
	Hepatology	2 (1.3)
	Other	23 (15.1)
Working in multiple areas of clinical practice	1 area	88 (57.9)
	2 areas	48 (31.6)
	3+ areas	16 (10.5)

Abbreviation: IQR, interquartile range.

^a^ Values are median (IQR).

^b^ Multiple answers permitted.

### 3.2 VLED/LED use within clinical practice

Of those surveyed, 109 (71.7%) reported currently using VLED/LED in clinical practice and 43 (28.3%) were not (Table 2). There was no difference in age or year of registration between participants that were using and not using VLED/LED. Of those using VLED/LED, the majority reported using them once or less per week (*n* = 92 [63.4%]), with only 14 (9.7%) used formula diets every day. Table 2 shows brands and types of diets used by participants, where Counterweight Pro800 and commercial meal replacements (e.g., Slim Fast; Tesco's Ultraslim) were the most popular (46.2% and 33.8% respectively).

**TABLE 2:** Very low‐energy diets and low‐energy diets utilization

Survey questions	
Do you currently use VLED/LED? (*n* = 152)	
Yes	109 (71.7)
No	43 (28.3)
How often do you use VLED/LED? (*n* = 145)	
One or less times a week	92 (63.4)
1–2 times per week	23 (15.9)
3–4 times per week	16 (11.0)
Everyday	14 (9.7)
What brands or types do you use with your patients?^a^ (*n* = 132)	
Counterweight Pro800	61 (46.2)
Commercial meal replacement	45 (33.8)
Optifast	14 (10.6)
Exante	18 (13.6)
Cambridge Weight Plan	8 (6.1)
Lighterlife	7 (5.3)
Food only	26 (19.7)
Milk only	17 (13.0)
Other	24 (18.2)
Using multiple brands	
1	82 (62.6)
2	25 (19.1)
3+	24 (18.3)
What patient population do you use formula diets with?^a^ (*n* = 129)	
People with overweight and obesity	69 (53.5)
People with T2D	67 (51.9)
People with T2D remission	75 (58.1)
People with fertility problems	20 (15.5)
People with orthopaedic problems	15 (11.6)
People who have undergone or undergoing bariatric surgery	28 (21.7)
Other	16 (12.4)
Working with multiple patient groups	
1	50 (38.8)
2	37 (28.7)
3+	42 (32.5)

Abbreviations: LED, low‐energy diet; T2D, type 2 diabetes; VLED, very low‐energy diet.

^a^ Multiple answers permitted.

Over half of the participants reported using VLED/LED to treat overweight or obesity, while 75 (58.1%) used them for T2D remission and 67 (51.9%) as part of T2D management. Other areas of practice such as fertility and orthopaedic problems were reported (Table 2). Further analysis identified those working in weight management and diabetes were more likely to use VLED/LED compared to those who were not using them for these purposes (*p* = .045; *p =* .015, respectively).

**FIGURE 1:**
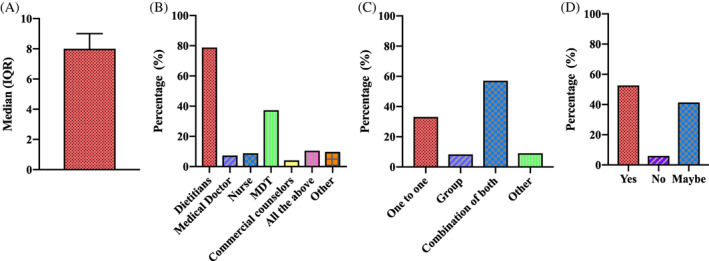
Responses to questions about dietitian's views and understandings towards very low‐ and low‐energy diets. (A) How would you rate your understanding of VLED/LED for treatment of obesity and T2D?^1^ (score out of 10); (B) Who should deliver VLED/LED?^2^; (C) What is the best way to deliver VLED/LED?^2^; (D) Do you believe that VLED/LED can achieve long‐term weight loss? LED, low‐energy diet; MDT, multidisciplinary team; T2D, type 2 diabetes; VLED, very low‐energy diet. Note: ^1^Median (IQR); ^2^multiple answers permitted

Participants reported their understanding of using VLED/LED for treatment of obesity and T2D was high, with a median score of 8.0 out of 10 (IQR 7, 9) (Figure 1A). Comparison between the areas of practice that dietitians work in revealed that higher understanding scores were associated with participants working in T2D, T2D remission and fertility problems (*r*_*s*_ = 0.250; *p* = .01; *r*_*s*_ = 0.287; *p* = .003; *r*_*s*_ = 0.193; *p* = .047; respectively), while working in bariatric surgery was negatively associated with higher understanding (*r*_*s*_ = −0.337; *p* < .0001). In addition, using Counterweight Plus Pro800 products was associated higher understanding of using VLED/LED for the management of obesity and T2D (*r*_*s*_ = 0.259, *p* = .007).

Most participants (*n* = 97 [78.9%]) reported that dietitians should be the ones delivering VLED/LED, while 37.4% (*n* = 46) reporting a multidisciplinary team (MDT) approach should also be used (Figure 1B). Furthermore, participants felt that the best mode of delivery was through a combination of one to one and group delivery (*n* = 69 [57%]), while a third of participants (*n* = 40 [33.1%]) reported one to one delivery alone was the preferred method (Figure 1C).

When asked whether long‐term weight loss was achievable following a VLED or LED, over half (52.6% [*n* = 61]) believed it was achievable, while only 6.0% (*n* = 7) believed it was not possible, with the remaining participants (*n* = 48 [41.4%]) unsure (Figure 1D). Participants not currently using VLED and LED were 3.48 times more likely to report that long‐term weight loss was not possible (*p* = .015) and were less likely to report greater understanding than those that currently using them (OR 0.37, 95% CI 0.16, 0.83; *p* = .016).

### 3.3 Attitudes and experiences of using formula VLED/LED

#### 3.3.1 Total diet replacement

Most participants (71.6% [*n* = 83]) reported using a TDR phase, in comparison to 28.4% (*n* = 33) who did not. Most dietitians reported that the TDR phase should last 12 weeks (*n* = 105 [89.7%]), with 57.5% (*n* = 69) reporting that patients should be seen every 2 weeks during this phase (Table 3).

**TABLE 3:** Responses of dietitians to questions about total diet replacement

Question	
Do you use TDR stage? (*n* = 116) (*n*, [%])	
Yes	83 (71.6)
No	33 (28.4)
How long on average do you think the TDR phase should last? (*n* = 117) (*n*, [%])	
4 weeks	10 (8.5)
8 weeks	24 (20.5)
12 weeks	71 (60.7)
16 weeks	2 (1.7)
20 weeks	4 (3.4)
Other	6 (5.1)
How often do you feel a patient should be seen within the TDR phase lasting 12 weeks? (*n* = 120) (*n*, [%])	
Every week	29 (24.2)
Bi‐monthly	69 (57.5)
Monthly	10 (8.3)
Other	12 (10.0)

Abbreviation: TDR, total diet replacement.

#### 3.3.2 Food reintroduction

Participants suggested the median duration of food reintroduction should last 8 weeks (IQR 6, 12), with responses ranging from 2 to 52 weeks. Approximately half of the participants (*n* = 64 [53.3%]) reported that patients should also be seen every 2 weeks during the food reintroduction phase (Table 3).

#### 3.3.3 Weight loss maintenance including rescue packages

When asked about the duration of the weight loss maintenance phase, participants suggested a median duration of 10.5 months (IQR 6, 12), with 74.8% (*n* = 89) reporting patients should be seen monthly during this time (Table 4). Rescue packages to assist weight regain were viewed as a popular option by participants (72.6% [*n* = 90]), with participants reporting that patients would need to gain a median of 3.0 kg (IQR 2, 4) to trigger their use. Participants viewed the inclusion of behavioural support and patient contact as the top two strategies (67.8% and 60.2%, respectively) for achieving long‐term weight loss maintenance.

**TABLE 4:** Dietitian's views and knowledge towards food reintroduction and weight maintenance

Question	
How often do you feel patients should be seen during the food reintroduction phase? (*n* = 120) (*n*, [%])	
Weekly	29 (24.2)
Bi‐monthly	64 (53.3)
Monthly	21 (17.5)
Other	6 (5.0)
How long on average do you think food reintroduction phase should be? (*n* = 102) weeks (median, [IQR])	8 (6–12)^a^
How often do you feel patients should be seen within weight maintenance phase? (*n* = 119) (*n*, [%])	
Weekly	1 (0.8)
Bi‐monthly	18 (15.1)
Monthly	89 (74.8)
3‐monthly	10 (8.4)
Other	1 (0.8)
How many months do you feel the weight maintenance phase should last? (*n* = 96) months (median [IQR])	10.5 (6–12)^a^
Should rescue packages be offered? (*n* = 124) (*n*, [%])	
Yes	90 (72.6)
No	16 (12.9)
Other	18 (14.5)
If yes, how much weight should a patient have regained before this is actioned? (*n* = 92) kg (median [IQR])	3.0 (2–4)^a^
What do you believe is essential for long‐term weight loss maintenance?^b^ (*n* = 118) (*n*, [%])	
Patient contact	71 (60.2)
Behaviour support	80 (67.8)
Continued use of meal replacement	10 (8.4)
Pharmacotherapy	4 (3.4)
All the above	34 (28.8)
Other	12 (10.2)

Abbreviation: IQR, interquartile range.

^a^ Values are median (IQR).

^b^ Multiple answers permitted.

#### 3.3.4 Motivation and confidence in implementing formula diets

To further understand dietitians' use of VLED/LED, participants were asked to score their motivation and confidence to implement them as part of clinical practice (Table 5). The vast majority reported having a high motivation score (7 out of 10; *n* = 94 [81.1%]), while over two thirds (*n* = 79 [68.8%]) reported high confidence score (7 out of 10) to implement VLED/LED in clinical practice.

**TABLE 5:** Motivation and confidence of dietitians for implementing very low‐ and low‐energy diet interventions within clinical practice

Survey question	
Motivation level in implementing a VLED/LED intervention within clinical practice (*n* = 116) (median [IQR])	8.0 (7, 10)
Confidence level in implementing a VLED/LED intervention within clinical practice (*n* = 115) (median [IQR])	8.0 (6, 9)

Abbreviations: IQR, interquartile range; LED, low‐energy diet; VLED, very low‐energy diet.

Further analysis showed that participants not using VLED/LED in practice were less likely to report being motivated (OR 0.26, 95% CI 0.11, 0.61; *p* = .002) or being confident (OR 0.08, 95% CI 0.03, 0.22; *p* < .0001) in implementing VLED/LED in clinical practice. While older participants were more confident about implementation (OR 1.08 per year increase in age, 95% CI 1.01, 1.16; *p* = .024). Furthermore, participants that used VLED/LED less than 3 times per week had a lower likelihood of reporting being highly motivated and confident to implement VLED/LED in clinical practice. While, using them once or less a week or once to twice a week had lower likelihood of being motivated (OR 0.16, 95% CI 0.04, 0.65; *p* = .011; OR 0.16, 95% CI 0.04, 0.73; *p* = .019, respectively) and confident of implementation (OR 0.12, 95% CI 0.03, 0.45; *p* = .001; OR 0.19, 95% CI 0.05, 0.79; *p* = .022, respectively).

#### 3.3.5 Barriers in implementing formula diets in clinical practices

**FIGURE 2:**
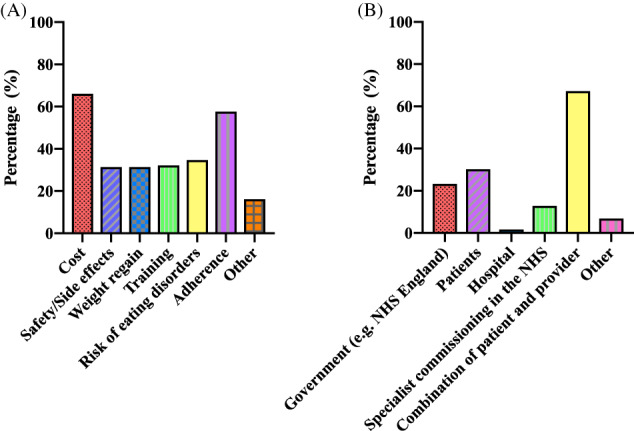
Factors affecting the use of formula very low‐ and low‐energy diets in clinical practices. (A) What barriers are there to you using formula diets in clinical practices?^1^; (B) Who should bear the cost of the products?^1^ Note: ^1^Multiple answers permitted

Dietitians identified two key barriers to implementing formula VLED/LED in practice: first cost (66.1% [*n* = 78]) and second adherence (57.6% [*n* = 68]) (Figure 2A). While two thirds (67.2% [*n* = 78]) reported the cost of formula VLED/LED products should be shared between the patient and the service provider (Figure 2B).

The belief that weight regain was a barrier to using formula VLED/LED was negatively associated with both motivation and confidence (*r*_*s*_ = −0.255; *p* = .006; *r*_*s*_ = −0.239; *p* = .010, respectively). While adherence being a barrier was associated with lower motivation (*r*_*s*_ = −0.238; *p* = .010) and concerns about side effects/safety being associated with lower confidence (*r*_*s*_ = −0.292; *p* = .002). Furthermore, there was a positive association between the frequency of using VLED/LED in clinical practice and belief that cost was a barrier to using VLED/LED (*r*_*s*_ = 0.193; *p* = .036).

#### 3.3.6 Qualitative analysis

Three key overarching themes were identified from the open‐ended survey responses: (1) perceptions of effectiveness, (2) level of intervention and (3) education and practice, with several subthemes grouped under each.

##### Theme 1: Perceptions of effectiveness

Overall, dietitians viewed formula VLED/LED interventions as an effective treatment for weight loss and T2D remission. At the same time, dietitians recognized that formula VLED/LED were a beneficial option for some patients, but were not suitable for everybody, and those using VLED/LED required substantial support including dietetic input. Dietitians reported that patient's motivation appeared to be a key factor to the success of the diets and acknowledged the need for the inclusion of the wider MDT in delivering VLED/LED interventions, in particular the role of health psychology.

*A very important option for individuals who want to lose weight and/or achieve diabetes remission. Achieving weight loss and diabetes remission is not a ‘one size fits all’ therefore having options are essential and increase likelihood of compliance*. Participant 101

*It has a place in clinical practice but must be used carefully, by experts and within an MDT with access to psychology*. Participant 9

*I think they need to be carefully supervised to prevent weight re‐gain. Weight loss and adherence to the diets is generally good but intensive follow up and transition to a normal healthy diet with a dietitian's support is essential*. Participant 74

*Work very well with highly motivated individuals*. Participant 129

The majority of the dietitians surveyed identified that the reintroduction of food following the TDR phase was challenging for patients and viewed the need for additional support and guidance from dietitians as crucial.

*An excellent tool for helping motivated patients improve health and achieve T2DM remission. Relatively simple intervention, although food reintroduction presents numerous challenges which a structured and supportive approach can help overcome*. Participant 4

##### Theme 2: Level of intervention

Dietitians typically viewed formula VLED/LED as a short‐term intervention, in order to achieve rapid weight loss, T2D remission or as a pre‐bariatric surgery intervention for ‘liver shrinkage’.

*They have their place for the right individual, who understands that this is a short‐term measure and is motivated to continue on to the 2nd phase of food reintroduction*. Participant 3

Some dietitians raised concerns around the long‐term safety of formula VLED/LED, risk of developing eating disorders and lack of evidence for long‐term outcomes.

…*I worry about the potential for VLCD to increase disordered eating and binge eating disorder*. Participant 134

*Happy to use them provided people have been adequately screened for eating disorders*. Participant 148

*Dangerous, harmful, promoting disordered eating*. Participant 151

*They work short term (e.g., pre‐op) but are challenging for some patients to stick to and query the long term effectiveness (without Bariatric surgery) in terms of maintenance of and significance of lost weight. In bariatrics pre‐op they appear effective in reducing some weight (reducing liver size) but am unsure of the overall significance per available data*. Participant 103

##### Theme 3: Education and practice

Some dietitians identified there were gaps in their training and education on the use of formula VLED/LED interventions, with the lack of delivery protocols resulting in limited or reluctant use of formula VLED/LED in practice. In contrast, those dietitians delivering the Counterweight‐Plus LED intervention reported a model of care that addressed the issues of training by providing ongoing education and support. There was also a sense that supervision or mentorship was needed to help with greater implementation within practice.

*Adequate training and ongoing support if required*. Participant 61

*We use the Counterweight Plus programme which is very robust with clear guidance on implementation in clinical practice and excellent support from the Counterweight team for medical queries etc*…. Participant 121

*Training, booklets, peer supervision and contact with questions (senior dietitian or specialist team)*. Participant 67

Dietitians reported differing funding arrangements for the implementation of formula VLED/LED programmes across the United Kingdom, e.g., central funding, or local commissioning. The lack of funding and guidance for commissioning formula VLED/LED interventions were consistently reported as a barrier to formula VLED/LED delivery. Dietitians also viewed the lack of infrastructure to support service delivery, e.g., clinic space/facilities, administrative support, formula diet product, lack of diverse resources and dietetic capacity, as a key implementation barrier. Furthermore, short‐term funding was reported as an obstacle to expanding formula VLED/LED provision where they were already being delivered.

*Commissioner guidance and funding, protocol for medical management in primary care*. Participant 128

*Time, staff, venue cost, primary care buy in (for weight management), cost sharing, printed info, data clerk*. Participant 116

*Time, evidence‐based programme, agreed process for funding of product, clinic space, funding and peer support*. Participant 38

*Resources need to accommodate a more diverse population, including meal plans for individuals or family based meals once food reintroduction is achieved*. Participant 147

## 4 DISCUSSION

Our study is the first in the United Kingdom to explore dietitians' views and experiences of using formula VLED/LED and showed that the majority of dietitians surveyed were currently using formula VLED/LED in clinical practice. This suggests a considerable number of clinical dietetic services in the United Kingdom are now using these dietary approaches particularly within weight management and diabetes services, which is in contrast to previous evidence showing VLED/LED are rarely used by HCP or dietitians.^12^, ^13^ Most dietitians felt VLED/LED were effective in some patients but not all, with patient motivation reporting to be a key aspect of success with VLED/LED interventions.

There was a strong sense from participants that dietitians should be core members of the delivery team of VLED/LED, with an effective MDT also being important, which reflects advice from current National Institute for Health and Clinical Excellence (NICE) guidance.^14^ Furthermore, it was acknowledged that clinical psychology was considered an essential part of the MDT to help support long‐term weight loss and adherence, despite there being limited access to psychology in current practice.^15^ This suggestion is likely to be due to multiple factors including reflection of national guideline for specialist service provision,^15^ also, evidence is emerging for the role of third‐wave cognitive therapies, e.g., acceptance and commitment therapy and their benefit for both weight loss and weight loss maintenance.^16^, ^17^ Dietitians also felt that a lack of effective infrastructure to help support service delivery, including facilities, administrative support and capacity, meaning that using VLED/LED was negatively impacted. These points highlight the importance of ensuring that VLED/LED programmes have the access to the appropriate staffing and support services prior to commencing to ensure long‐term success which may present a challenge for NHS services to effectively incorporate VLED/LED programmes without additional funding.

Our data show a lack of agreement from dietitians on how VLED/LED should be delivered within practice, with many services appearing to be replicating how research studies have delivered their programmes in terms of intensity and timing,^5^, ^18^, ^19^ The food reintroduction phase was reported to be the most challenging phase by dietitians, which agrees with evidence from qualitative evaluations that have also identified the food reintroduction phase as the most challenging for patients^20^ and highlights the need for additional dietetic support during the crucial phase. The use of ‘rescue packages’ for preventing or treating weight regain was popular with dietitians in our survey. The mean threshold for weight regain before the use of ‘rescue packages’ should be considered was suggested as 3 kg compared to 2 kg within DiRECT.^21^, ^22^

The updated review of the NICE obesity guidelines in 2014^14^ recommended that VLED should not be used routinely within clinical practice, could be used for no longer than 12 weeks and required specialist input. These recommendations may have in part impacted the decision and confidence of UK dietitians to implement VLED within practice. However, it should be noted that the guidelines only included research studies up to the year 2000 therefore excluded high‐quality studies for the use of VLED between 2000 and 2014,^23^, ^24^ and furthermore only focused on VLED and did not include evidence for LED, which are now more commonly used in research trials and clinical practice. Therefore the recently published high‐quality studies over the last decade were omitted demonstrating their effectiveness^5^, ^18^, ^19^ and there have been suggestions there is a need for a review of the NICE recommendations^4^ to help better guide practice.

The barriers to implementation of VLED/LED by dietitians appeared to be driven mainly from beliefs about cost and a lack of adherence, alongside a lack of funding and guidance for commissioning, Data supports the view that cost is a barrier to wider use,^25^ with patients having to self‐fund being identified as a particular barrier to continuation of the programme,^26^ although self‐funding was not mentioned specifically as an issue by the participants. However, in comparison when looking at adherence, the views held by dietitians appear contrary to the available evidence. With systematic review data comparing VLED against control (food‐based low‐energy/fat diet) finding no difference in levels of adherence,^4^ which may suggest that no matter what diet patients choose, adhering to it will be challenging. Furthermore, qualitative data suggests that although people initially found transitioning to a VLED/LED challenging, it quickly eased, was easier than expected and they enjoyed the simplicity of the VLED/LED, which all facilitated adherence.^26^, ^27^, ^28^ Also, a recent both narrative and systematic review has shown VLED/LED to be the most effective treatment for T2D remission.^25^, ^29^

Motivation and confidence have been suggested to play key roles in determining human behaviour, with confidence indicating one's ability to perform the behaviour and motivation their desire to engage in that behaviour.^30^ Within this study, we measured both in relation to belief about implementation of VLED/LED with people living with obesity and T2D and showed a higher degree of both was present in dietitians currently using them in practice compared to those that were not and dietitians infrequently using VLED/LED negatively impacting their motivation and confidence in implementation. Taken together these associations might in part explain why some dietitians use VLED/LED and others do not, and might suggest that in order to help increase implementation that education and training to help increase motivation and confidence could be key.

Dietitians typically view formula VLED/LED as short‐term interventions, which reinforces the view that following weight loss using VLED/LED weight regain is inevitable.^31^, ^32^ This historical view may well in part be driving dietitian's beliefs that these diets are not suitable for achieving weight loss maintenance and limiting their implementation and is felt by other HCP as a key reason for not using meal replacements.^13^ Despite this, over half the dietitians felt that long‐term weight loss was achievable, which is supported by data showing that with long‐term follow‐up and continued use of formula products that weight loss maintenance is achievable, even in those with osteoarthritis.^8^, ^33^ However, within practice maintenance is rarely achieved, due to long‐term support being seldom provided or included in guidelines or clinical practice.^14^, ^34^

One key benefit of using VLED/LED is the degree of weight loss, with data showing on average people lose over 10%,^5^, ^18^, ^19^ which has been shown to be associated with long‐term weight maintenance.^35^, ^36^, ^37^ Despite this, few services achieve this degree of weight loss with weight losses in UK Tier 3 weight management service ranging between 3 and 6 kg.^38^ This might suggest that services would benefit from changing their practices to include the use of VLED/LED as part of usual care instead of less efficacious lifestyle interventions. Firstly, this would be more in line with the weight loss expectations of people living with obesity, with expectations of 11%–34% being reported,^39^ and secondly more likely to achieve greater health improvements and weight maintenance.^5^, ^40^, ^41^

Interestingly, the risk of developing an eating disorder as a barrier for implementation of VLED/LED was expressed by over a third of dietitians and infrequently mentioned in the open‐ended questions, although some felt very strongly that VLED/LED should not be used at all due to the potential risk as seen in the quotes in Section 3. Systematic review data, however, does not support this view^42^ with further evidence showing a 50% reduction in participants meeting DSM‐IV criteria for binge eating disorder 12 months after using a VLED programme.^43^ This would suggest that although caution should be taken to minimize risk prior to commencing VLED/LED, including adequate screening, and monitoring for eating disorders, it should not be viewed as a contraindication to use in relation to binge eating disorder. It should be noted that data exploring the use of VLED and other eating disorders within people living with overweight or obesity is lacking.

Those dietitians surveyed that were using the Counterweight‐Plus programme appeared to have a greater understanding of using VLED/LED for the management of obesity and T2D. This is likely to be related to both the educational element added to the delivery of their programme and ensuring that dietitians using their programme have continued clinical mentorships, which has been reported by the dietitians using the Counterweight‐Plus programme^5^, ^44^ and also within our data. Education has been shown to be important, with recent data identifying that those with formal education on using VLED/LED had an increased likelihood of using meal replacement products.^13^ Data from our qualitative analysis showed similar results^13^ and identified that dietitians acknowledged gaps in their training and education around the use of VLED/LED resulting in a reluctance to use them in practice. This suggests if VLED/LED interventions are going to be used more extensive at a national level then training and education programmes should be developed and be a key consideration in the upcoming low‐calorie diet pilot planned by NHS England.^9^

Safety and side effects have historically been an issue with VLED,^2^ although modern VLED/LED formulations, which meet EU guidelines, are now safe. The main side effects reported from the literature include alterations to bowel habits (e.g., constipation), light‐headedness, fatigue and hair loss.^5^, ^18^, ^19^, ^41^ Despite substantial evidence showing that side effects are transient, moderate and generally easily manageable,^5^, ^18^, ^19^, ^41^ dietitians in our study reported side effects as a barrier to implementation and resulting in lower confidence in their use, which is similar to other concerns voiced by other HCP.^13^ The exact reason why there is a discrepancy remains unknown, although this may relate to historical views regarding safety^2^ and therefore in order to address this incongruence further education appears key.

The main strength of this study is that it is the first study that has explored UK dietitians' attitudes and experiences of using VLED/LED in clinical practice. Another strength was the combination of both quantitative and qualitative data analysis and allowing a more extensive understanding of the issues around this important topic.^45^ There are also limitations to the current survey. First, the study was designed as a cross‐sectional study and is limited to the methods of recruitment used, meaning that we only have an understanding from those dietitians who had access to the survey link. Second, of those who were included, 46% of participants were currently using the Counterweight‐Plus programme, which may have in part impacted the outcomes of the survey particularly around motivation and confidence being so high and should be taken into consideration. Thirdly, during the recruitment process, we attempted to have a representative sample of dietitians including those working with people with eating disorders who may have traditionally opposed the use of VLED/LED. Despite this the sample from this group was small, therefore this should be acknowledged as a limitation of our study. Finally, it was not clear from our data whether patient motivation being important to success was an opinion held by dietitians about their patients or whether they had measured motivation. Therefore, it is important dietitians do a comprehensive assessment to gauge motivation directly to ascertain if motivation is an important factor to success when using VLED/LED or simply an opinion of the dietitians surveyed.

## 5 CONCLUSIONS

This novel study sheds new light on the attitudes and experiences of using VLED/LED held by dietitians in the United Kingdom. This expands our understanding of how current practices are working, and identifies key areas that need addressing if these dietary interventions are going to be embedded in practice. With clinical services now wishing to implement VLED/LED programmes, particularly to engender T2D remission, understanding and detailing the potential barriers and facilitators are essential for commissioners, service providers and clinicians. This study highlights the importance of training and education in the delivery of TDR programmes. It is therefore key that national educational programmes are developed to help increase confidence and understanding of how to effectively use and implement VLED/LED in a wide, diverse patient population living with obesity.

## CONFLICT OF INTEREST

Adrian Brown reports grants from Cambridge Weight Plan, outside the submitted work; and is the Vice‐Chair of Specialist Obesity Group of the BDA, on the Strategic Council for APPG Obesity and on the Medical Advisory Board and shareholder of Reset Health Clinics Ltd. Rachel L. Batterham reports personal fees from Novo Nordisk, other from Novo Nordisk, personal fees from Pfizer, personal fees from International Medical Press, personal fees from Boehringer Ingelheim, personal fees from ViiV, from null, outside the submitted work. Stuart W. Flint reports grants from Johnson & Johnson, grants from Novo Nordisk, personal fees from Novo Nordisk, outside the submitted work. Naomi Brosnahan has received funding for PhD fees and conference attendance from Cambridge Weight Plan Ltd, and is a current employee and shareholder in Counterweight Ltd. Dorsa Khazaei and Jed Wingrove have no conflicts.

## AUTHOR CONTRIBUTIONS

Adrian Brown conceived the study. Adrian Brown, Naomi Brosnahan and Rachel L. Batterham contributed to the study and survey design and methodology. Adrian Brown was responsible for the oversight of the study. Adrian Brown and Naomi Brosnahan contributed to the recruitment of participants. Adrian Brown, Naomi Brosnahan, Dorsa Khazaei, Jed Wingrove and Stuart W. Flint were responsible for the data analysis. All authors contributed to data interpretation and the writing of the manuscript. All authors contributed to the critical revision of the manuscript and gave final approval.

## Supporting information


**Appendix** **S1**: Supporting Information.
