# Supplementary material for: UK dietitians' attitudes and experiences of formula very low‐ and low‐energy diets in clinical practice
Source: Clin Obes. 2022 Jan 23;12(3):e12509. doi: 10.1111/cob.12509 (PMC9286801; doi:10.1111/cob.12509)
Supplement: Supplementary file 1 — Appendix S1: Supporting Information. [file COB-12-0-s001.docx]

**Supplementary Material**

**Survey Questions:**

**Demographics**

Are you a registered dietitian? 1) Yes 2) No

Years since registration - state

Age - state

Gender 1) male 2) female 3) transgender 4) other 5) prefer not to say 6) other

Country of residency – 1) England 2) Scotland 3) Wales 4) Northern Ireland 5) Ireland 6) Other

**Background**

1. Which area of clinical practice do you current work in? 1) weight management 2) diabetes 3) Bariatric Surgery 4) Endocrinology 5) Hepatology 6) Paediatrics 7) Other: please name
2. Do you currently use formula low or very low energy diet? 1) yes 2) no
3. How often do you use formula diets? 1) 1 or less a week 2) 1-2 times a week 3) 3-4 time a week 4) everyday
4. Which brand or type do you use with your patients? 1) Lighterlife 2) Optifast 3) Cambridge Weight Plan 4) Counterweight Pro800 5) Exante 6) commercial meal replacements e.g. Slimfast; Tesco’s Ultraslim 7) Food only 8) milk only 8) other give details
5. What patient populations do you use formula diets with? 1) people with overweight and obesity 2) Type 2 diabetes remission 3) People with type 2 diabetes 4) People with fertility problems 5) People with orthopaedic problems 6) Patients who have undergone or undergoing bariatric surgery 7) other…please name

**Views and Experiences**

1. What is your feelings about VLED/LED within clinical practice? Open ended question
2. How would you rate your understanding of VLED/LED for the treatment of obesity and T2D - VAS
3. What’s the best way to deliver a VLED/LED programme 1) 1:1 2) group 3) combination of both 4) other give details
4. Do you use a total diet replacement stage (i.e. period using only formula product, TDR) 1) yes 2) no 3) other give details
5. How long on average do you think the TDR phase should be? 1) 4 weeks 2) 8 weeks 3) 12 weeks 4) 16 weeks 5) 20 weeks 6) other give details
6. How often do you feel patient should be seen within the TDR phase lasting 12 weeks? 1) every week 2) bi-monthly 3) monthly 4) other
7. How long on average do you think the food reintroduction phase should be? Open ended question
   1. How often do you feel patient should be seen within the food reintroduction phase? 1) every week 2) bi-monthly 3) monthly 4) other
8. How many months do you feel the weight maintenance phase should last?
   1. How often do you feel patient should be seen within the weight maintenance phase? 1) every week 2) bi-monthly 3) monthly 4) other
9. Who should deliver the VLED/LED 1) dietitian 2) medical doctor 3) nurse 4) Multidisciplinary Team 5) commercial counsellors 6) all the above 7) other give details
10. Should rescue packages be offered (i.e. if a patient regains weight to return to a total or partial diet replacement for period of time) 1) Yes 2) No 3) other
    1. If yes, what percentage of total weight loss (%) should a patient have regained before this is actioned?

**Barriers**

1. What barriers are there to you using formula diets in clinical practice? 1) cost 2) side effects/safety 3) weight regain 4) training 5) risk of eating disorders 6) adherence 8)other give details
2. Do you believe that VLED/LED can achieve long term weight loss 1) Yes 2) No 3) maybe
3. How motivated are you to implement a VLED/LED diet intervention within clinical practice? Visual Analogue scale from 1-10
4. How confident are you to implement a VLED/LED diet intervention within clinical practice? Visual Analogue scale from 1-10
5. Who do you think should bear the cost of the product 1) Government in country residing (e.g. NHS England, NHS Scotland) 2) Patient 3) Hospital 4) Specialist Commissioning in NHS 6) combination of patient and provider - subsidised 7) other

**Facilitators**

1. What resources do you feel you would need to implement a VLED intervention? Open ended question
2. What do you believe is essential for long term weight loss maintenance? 1) patient contact 2) behavioural support 3) continued use of meal replacement 4) pharmacotherapy 5) all the above 6) other please explain
3. Is there anything else you would like to comment on that hasn’t been covered by the abover questions? Open ended question
